# Managing functional disorders: opportunities and threats

**DOI:** 10.1080/02813432.2020.1718304

**Published:** 2020-02-26

**Authors:** Helena Liira

**Affiliations:** Scandinavian Journal of Primary Health Care Adjunct Professor, General Practice, University of Helsinkie

Approximately, 10–15% of patients treated by general practitioners (GPs) consult because of symptoms that cannot be easily diagnosed with existing diagnostic coding systems. These symptoms have been called ‘medically unexplained physical symptoms’, ‘MUPS’ or ‘MUS’, and especially in North America as ‘somatic symptom disorders’.

The Research Clinic for Functional Disorders and Psychosomatics in Aarhus, Denmark is a GP led clinic that pursues a research agenda in this field. It has applied the terms ‘functional disorders’, ‘health anxiety’ and ‘bodily distress syndrome’, which is also a new ICD-11 code. A lot of criticism from patient organizations has been focused on the term ‘functional disorders’ and all psychological treatments for them. The criticism stems from some patient representatives still believing these disorders to have a solely biological origin. Chronic fatigue syndrome (CFS) and its causes are particularly debated.

However, an increasing amount of research evidence exists to support the term ‘functional’ instead of medically unexplained [1,2]. These disorders most likely have a multifactorial origin, including genetic, predisposing and triggering factors. Stress is prevalent [3], and even toxic stress experienced in childhood is linked to these conditions [4]. The central mechanism in functional disorders appears to be a sensitization of the central nervous system, which can cause a multitude of symptoms from the overactive autonomous nervous system [2]. Recent imaging studies that used functional MRI, support this theory [1].

A worrying phenomenon is that researchers working on functional disorders, especially CFS, face harsh online abuse and harassment. Like many leading scientists, Michael Sharpe, an Oxford based psychologist, quit the CFS research last year saying that the field had become ‘too toxic’ [5]. Guidelines editors and health policy makers have experienced similar campaigning from the CFS community.

Another threat is the increasing shortage of continuity of care in general practice. In Denmark, the number of GPs decreases while population ages and the demands increase [6]. Similarly, in Norway, there is an ongoing recruitment crisis of GPs [7]. In Finland, a health care reform has been attempted for 15 years to improve the suffering primary health care, without success. Continuity of care and the context of general practice are crucial for diagnostic knowing [8], a basic skill needed with the complex functional disorders.

A third challenge relates to potential medical overuse. Patients with functional disorders cause diagnostic challenges and often experience excessive diagnostic testing, which is another and costly threat in their management. Medical overuse is a dilemma for GPs, and they understand the potential harm it may cause to patients [9]. Excessive testing, uncertainty and negative expectations cause harmful nocebo effects that can develop into somatic symptoms and anxiety [10].

On the other hand, the placebo-nocebo research provides new insights into the patient encounter [11]. With functional disorders, explaining the nocebo effects may help patient understand the worsening of symptoms. Focusing less on symptoms and more on cure reduces the nocebo effects. At the same time, the placebo effects such as trust, hope, continuity of care and good communication form essential parts of the cure.

Finally, the term ‘functional’ may also entail optimism. It means that no permanent injury has taken place and that the disorder is reversible. The sensitized central nervous system may be retrained. To help educating patients in this process, there are useful Internet resources such as the site created by the Scottish neurologist Jon Stone: neurosymptoms.org. A patient organization, FND Hope, empowers and supports patients with functional neurological disorders.

There is evidence from a Cochrane review that cognitive behavioral therapy (CBT) reduces somatic symptoms [12]. However, CBT is not widely available and modifications suitable for large groups of patients are necessary. Psychoeducation in groups can alleviate the symptoms in these patients [13]. Web-based, guided self-help is another promising alternative [14]. Nevertheless, general practice needs courageous researchers and further development of interventions to tackle the burden of illness by functional disorders.
